# Variance heterogeneity analysis for detection of potentially interacting genetic loci: method and its limitations

**DOI:** 10.1186/1471-2156-11-92

**Published:** 2010-10-13

**Authors:** Maksim V Struchalin, Abbas Dehghan, Jacqueline CM Witteman, Cornelia van Duijn, Yurii S Aulchenko

**Affiliations:** 1 Department of Epidemiology, Erasmus MC, Rotterdam, 3000 CA, The Netherlands; 2 Quantitative Integrative Genomics Group, Institute of Cytology and Genetics SD RAS, Novosibirsk, 630090, Russia

## Abstract

**Background:**

Presence of interaction between a genotype and certain factor in determination of a trait's value, it is expected that the trait's variance is increased in the group of subjects having this genotype. Thus, test of heterogeneity of variances can be used as a test to screen for potentially interacting single-nucleotide polymorphisms (SNPs). In this work, we evaluated statistical properties of variance heterogeneity analysis in respect to the detection of potentially interacting SNPs in a case when an interaction variable is unknown.

**Results:**

Through simulations, we investigated type I error for Bartlett's test, Bartlett's test with prior rank transformation of a trait to normality, and Levene's test for different genetic models. Additionally, we derived an analytical expression for power estimation. We showed that Bartlett's test has acceptable type I error in the case of trait following a normal distribution, whereas Levene's test kept nominal Type I error under all scenarios investigated. For the power of variance homogeneity test, we showed (as opposed to the power of direct test which uses information about known interacting factor) that, given the same interaction effect, the power can vary widely depending on the non-estimable direct effect of the unobserved interacting variable. Thus, for a given interaction effect, only very wide limits of power of the variance homogeneity test can be estimated. Also we applied Levene's approach to test genome-wide homogeneity of variances of the C-reactive protein in the Rotterdam Study population (*n *= 5959). In this analysis, we replicate previous results of Pare and colleagues (2010) for the SNP rs12753193 (*n *= 21, 799).

**Conclusions:**

Screening for differences in variances among genotypes of a SNP is a promising approach as a number of biologically interesting models may lead to the heterogeneity of variances. However, it should be kept in mind that the absence of variance heterogeneity for a SNP can not be interpreted as the absence of involvement of the SNP in the interaction network.

## Background

Genome-wide association (GWA) study has become the tool of choice for the identification of loci associated with complex traits. In GWA analysis, the association between a trait of interest and genetic variation is studied by using thousands of subjects typed for hundreds of thousands of polymorphisms. Thus several hundred loci for dozens of complex human disease and quantitative traits have been discovered utilizing this method [1].

However, it has become clear that for most complex traits, loci discovered using GWA studies currently explain a small portion of total trait's heritability and are not likely to explain all of the heritability of the trait even with additional new loci discovered using progressively larger sample sizes [2,3]. A number of strategies that may help discovering the sources of this "missing heritability" have been suggested [4]. In particular, it was suggested that exploring more complex genetic models, such as these accounting for gene-gene (epistatic) and gene-environment interactions is a promising approach. In the context of genetics, interactions refer to a phenomenon when the effect of an allele at a particular locus changes given the value of another (interacting) factor, which may be another allele at the same locus (e.g. dominance inter-locus interactions), or alleles at other loci (epistasis) or some other factor (end- or exogenous environment). However detection of epistatic and gene-environment interactions is a challenging task. In GWA scans, millions of SNPs are typed and imputed [5]. Compared to standard analysis of marginal effects, a direct search for pairs of interacting loci roughly squares the number of tests to be performed making this task both computationally and methodologically difficult. A search for gene-environment interaction, unless there are a *priory *evidence that particular environmental factor is highly likely to interact with genotype, involves search of the interacting environmental factor throughout the environmental and phenomic space, again, increasing the number of tests to be performed, and leading to computational and methodological challenge.

If a method allowing detection of SNPs potentially involved in interaction networks based on the SNP and trait information (but not the information about the interacting factor(s)) existed, that would provide a substantial advancement to the field. Indeed, if such method existed, we could first screen potentially interacting SNPs using such method, and then restrict the search for the other interacting factor (genetic or environmental) to these SNPs only, dramatically decreasing the search space.

**Figure 1:**
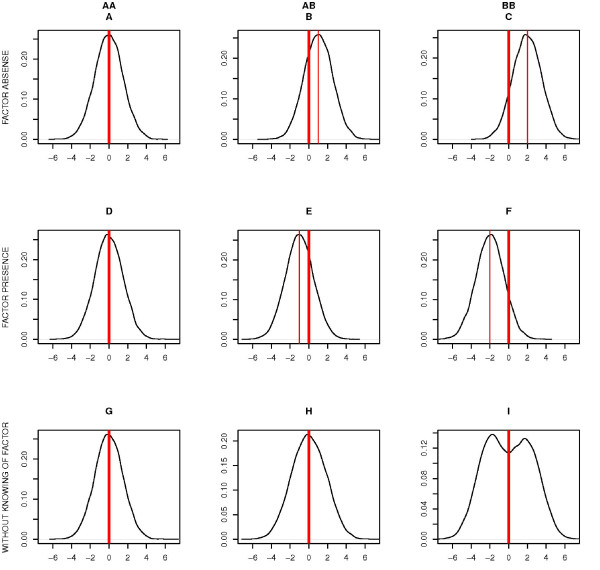
**Distribution of hypothetical trait with expectation determined by genotype and its interaction with a binary trait**. A, B, C: distribution of the trait for genotypes AA, AB, BB, correspondingly in a case when the interacting factor is present. D, E, F: distribution of the trait for genotypes AA, AB, BB, correspondingly in a case when the factor is absent. G, H, I: distribution of the trait for genotypes AA, AB, BB, correspondingly in a case when factor is unknown. In this case the distributions present mixtures of upper two ones.

It has been suggested that analysis of equality and heterogeneity of variances of the trait between different genotypes may become such a tool [6]. If a particular genotype is interacting with some (yet unknown) factor, it could modify the marginal mean (computed from the model not including the interactor) of a trait of subjects having this genotype, and it will also increase the marginal variance of the trait: in effect the distribution of the trait in the group of subjects with interacting genotype will be described by a mixture of distributions with different means, leading to increased variance of the trait within this group. Figure 1 shows the distribution of a hypothetical trait in a case of a binary factor interacting with a SNP. The upper three plots show distribution of the trait for each genotype in case of presence of the factor. Three plots in the middle show distribution of the trait for each genotype in case of absence of the factor. The lower three plots show distribution of the trait for each genotype in case when the factor is unknown and distinguishing of subjects by the factor is impossible. Theses three plots are the mixture of the distributions from upper plots for each genotypes correspondingly.

In this work, we assume an underlying model, in which the trait is generated based on knowledge of the SNP genotype and the interacting factor, and using fixed assumed model parameters. The analysis of variances of the trait is based on SNP information only, as the interacting factor is assumed to be unknown in such analysis aimed to identify potentially interacting SNPs without knowledge of an interacting variable. Using this defined framework we first evaluate type I error of different variance heterogeneity tests using simulated data. Second, assuming known interaction model involving SNP and an interacting factor, we relate the power of the variance heterogeneity test to the parameters of the underlying model.

### Underlying model of the trait

We assumed the following linear model:

(1) $$y_{i}\sim \mu +\beta_{g}g_{i}+\beta_{F}F_{i}+\beta_{gF}\cdot g_{i}F_{i}+\epsilon_{i},$$

where *y*_*i *_is a value of the trait for *i*^*th *^individual, *μ *is intercept, *β*_*g *_is effect of a SNP, *β*_*F *_is effect of an interacting factor, *β*_*gF *_is effect of interaction between the SNP and the factor, *g*_*i *_~ *B*(*n*_*g*_, *P*_*B *_) is a SNP, which is assumed to be binomialy distributed with *n*_*g *_= 2 (number of alleles in the genotype) and *P*_*B *_∈ [0; 1] (frequency of the interacting *B *allele). Below the notation *AA*, *AB *and *BB *is used for indicating a genotype having zero, one and two interacting alleles *B *correspondingly. $F_{i}\sim N(\mu_{F},\sigma_{F}^{2})$ is a factor, which is assumed to be normally distributed with mean *μ*_*F *_and variance $\sigma_{F}^{2}$. ϵ_*i *_is residual random error. Since many traits regularly are not normally distributed we studied seven types of distribution of ϵ_*i*_: normal distribution, *t*-distribution (with *df *= 2, 5, 10) and *χ*^2 ^distributions (with *df *= 1, 5, 15). ϵ_*i *_was standardized to have zero mean and variance of one. We assumed that the distributions of *g*_*i*_, *F*_*i*_, and ϵ_*i *_are independent.

Without loss of generality we can assume that *μ *= *μ*_*F *_= 0, and $\sigma_{F}^{2}$ = 1.

### Homogeneity of variance tests

Bartlett's test is defined as:

(2) $$T^{2}=\frac{\left(N-k)ln(\sigma_{p}^{2})-\sum_{j=0}^{k-1}\left(n_{j}-1\right)ln(\sigma_{j}^{2}\right)}{1+\frac{1}{3(k-1)}\left(\sum_{j=0}^{k-1}\left(\frac{1}{n_{j}-1}-\frac{1}{N-k}\right)\right)},$$

where *k *is the number of genotypes tested, *n*_*j *_is the sample size of the *j*^*th *^group (*j *possess the integer values from zero to *k *- 1), $N=\sum_{j=0}^{k-1}n_{j}$ is the total sample size, $\sigma_{j}^{2}=\frac{1}{n_{j}}\sum_{i=1}^{N}{\left(y_{i}-{\bar{y}}_{j}\right)}^{2}I_{g_{i}=j}$ is variance of the *j*-th group, where *I*_*a*__=__*b *_is an indicator variable taking value one if *a *= *b *and zero otherwise. *y*_*i *_is a value of the trait for *i*_*th *_individual, *g*_*i *_is a SNP of *i*^*th *^individual, ${\bar{y}}_{j}=\frac{1}{n_{j}}\sum_{i=1}^{N}y_{i}I_{g_{i}=j}$ is mean value of the trait for group *j*, $\sigma_{p}^{2}=\frac{1}{N-k}\sum_{j=0}^{k-1}\left(n_{j}-1\right)\sigma_{j}^{2}$. Under a null hypothesis of variance homogeneity, the value of the test, *T*^2^, is distributed as $\chi_{df=(k-1)}^{2}$.

Bartlett's test with prior rank-transformation to normality was done by applying Bartlett's test to a transformed trait. Rank-transformation to normality is transformation (in absence of ties) that leaves the same ranks but distribution becomes perfectly normal.

Levene's (Brown-Forsythe) test is defined as:

(3) $$T^{2}=\frac{(N-k)\sum_{j=0}^{k-1}n_{j}{\left(Z_{j}.-Z..\right)}^{2}}{(k-1)\sum_{i=1}^{N}\left(Z_{i}-Z_{g_{i}}.\right)},$$

where $Z_{i}=|y_{i}-{\tilde{y}}_{g_{i}}|,{\tilde{y}}_{g_{i}}$ is median value of the trait for genotype *g*_*i*_, $Z_{j}.=\frac{1}{n_{j}}\sum_{i=1}^{N}Z_{i}I_{g_{i}=j}$, $Z..=\frac{1}{N}\sum_{i=1}^{N}Z_{i}$.

Under a null hypothesis of variance homogeneity, the value of the test, *T*^2^, is distributed as *F *with *df*_1 _= (*k *- 1) and *df*_2 _= (*N *- *k*) degrees of freedom. In our case, where *N *= 10,000, *T*^2 ^is excellently approximated with $\chi_{df=(k-1)}^{2}$

The number of genotypes is at the most three, which corresponds to genotypes *AA*, *AB *and *BB*. Thus, the variance homogeneity test results to a test with two degrees of freedom. We also considered three tests with one degree of freedom that test variance of a particular genotype against two others ( *AA *vs. *AB *and *BB*, *AB *vs. *AA *and *BB*, and *BB *vs. *AA *and *AB*). For those tests we reduced trait's distribution of each genotypes to zero mean.

### Simulations

To study Type I error, simulations were performed. Effects of a factor and an interaction term were set to zero (*β*_*F *_= *β*_*gF *_= 0). Interacting allele frequencies studied were set to 5%, 10%, 25%, and 50%. For each fixed allelic frequency, we set the effect of SNP, *β*_*g *_in order to explain 0%, 1%, and 5% of the total variance of the trait. Denoting this proportion as *r*^2^, the corresponding SNP effect was computed as

(4) $$\beta_{g}=\sqrt{\frac{r^{2}\sigma_{\epsilon }^{2}}{\left(1-r^{2})2P_{B}(1-P_{B}\right)}},$$

where $\sigma_{\epsilon }^{2}$ is variance of a residual error which was assumed to be one. Thus, for each from one and two degrees of freedom tests eighty four models were studied. For each model point we simulated data for 10,000 individuals, and simulations were repeated 10, 000 times.

Under the alternative hypothesis, assuming normally distributed residual error, we have developed an analytical expression for *NCP *(see subsection **Power **in section **Results**). To check correctness of our analytical solutions, we have studied several points from the model space by simulations. The parameters studied were allele frequency *P*_*B *_= {0.05, 0.5}, SNP effect *β*_*g *_= {0, 0.3}, and effect of factor *β*_*F *_= {0, 1}.

### Power of direct test for interactions

The difference in power between direct method and variance homogeneity tests were also studied. Direct test was defined as regression analysis when all variables, including the interacting factor, are known and relationships between dependent and independent variables are estimated.

Power is a function of non-centrality parameter. Analytical expression for non-centrality parameter (*NCP*) of test statistics to detect effect of interaction *β*_*gF *_by direct test is

(5) $$NCP=\beta_{gF}^{2}\frac{1}{\sigma_{\epsilon }^{2}}N\left(\sigma_{gF}^{2}-\frac{cov^{2}(F,g\cdot F)}{\sigma_{F}^{2}}\right),$$

Where $\sigma_{gF}^{2}=2P_{B}(1+P_{B})\sigma_{F}^{2},\;cov(F,g\cdot F)=2P_{B}\sigma_{F}^{2}$, is covariance between *F *and *g*·*F*.

## Results

### Type I error

**Figure 2:**
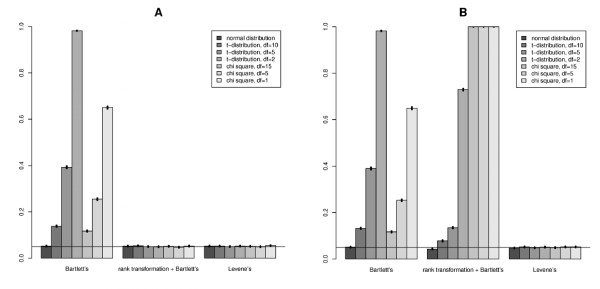
**Type I error at the threshold corresponding to *α *= 5% for interacting allele frequency 10%**. A: SNP effect is absent, B: SNP effect explains 5% of total trait's variance.

Figure 2 shows type I error rate obtained in our simulation study for different variance homogeneity tests. Type I error corresponds to the threshold *α *= 5% and interacting allele frequency 10%. Plot A shows the results for the model without SNP effect, whereas plot B represents results for the model with SNP effect explaining 5% of the total trait's variance. Each column presents one distribution of residual error, each group of columns represents one variance homogeneity test. For both figures, the interacting allele frequency *P*_*B *_= 10%.

From Figure 2, one can see that type I error of Bartlett's test grows with increase of asymmetry as well as with heavier tails of distribution.

Bartlett's test with prior rank transformation to normality has acceptable type I error 5% only in case of SNP effect absence. Only type I error of Levene's test does not show dependence on model parameters. In case of SNP effect presence, rank transformation to normality of a trait which follows a non-normal distribution results to perfectly normally distributed trait whereas distribution of a trait for each genotype becomes distorted. Additional file 1, Figure S1 shows distribution of a trait for each genotype before and after transformation in case of SNP effect presence, explaining 5% of total variance.

Results for type I error for other frequencies of interacting allele are similar to those shown in Figure 2. Additional file 2, Table S1, S2, and S3 present type I error in case there is SNP effect explaining correspondingly 0%, 1%, 5% of total traits's variance. Each of these tables present result for different interacting allele frequency *P*_*B *_= 5%, 10%, 25%, and 50%

Results for type I error for one degree of freedom tests are presented in the tables of Additional file 3, Additional file 4, and Additional file 5. The notable difference from two degrees of freedom test is that even in absence of SNP effect Bartlett's test with prior rank transformation of a trait has increased type I error.

### Power

We have derived an expression for dependence of trait's variances on model parameters for each genotype of a SNP.

$$\begin{array}{l} \sigma_{AA}^{2}=\beta_{F}^{2}\sigma_{F}^{2}+\sigma_{\epsilon }^{2}\\ \sigma_{AB}^{2}=\sigma_{AA}^{2}+\beta_{gF}^{2}\sigma_{F}^{2}+2\beta_{gF}\beta_{F}\sigma_{F}^{2}\\ \sigma_{BB}^{2}=\sigma_{AA}^{2}+4\beta_{gF}^{2}\sigma_{F}^{2}+4\beta_{gF}\beta_{F}\sigma_{F}^{2}, \end{array}$$

where $\sigma_{AA}^{2}$, $\sigma_{AB}^{2}$ and $\sigma_{BB}^{2}$ are variances of trait's distribution in each group of subjects having corresponding genotype.

**Figure 3:**
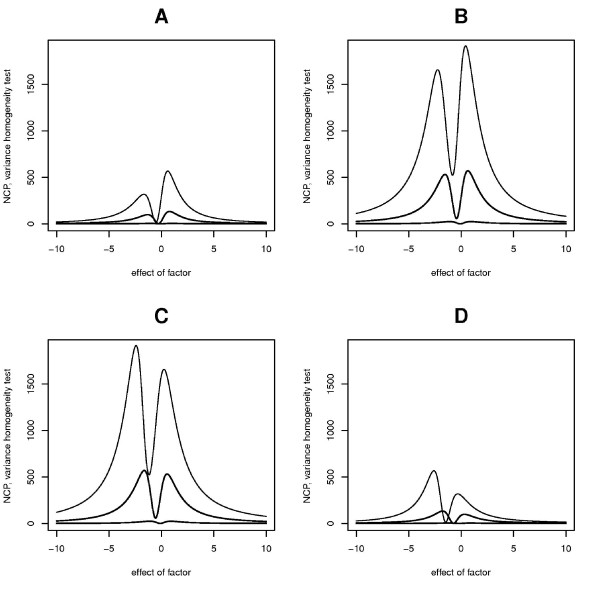
**Dependence of non-centrality parameter of variance homogeneity test on main effect of a factor**. The top curve on each plot shows results for interaction effect *β*_*gF *_= 1, the middle curve is for *β*_*gF *_= 0.5, and the bottom curve is for *β*_*gF *_= 0.1. Each subplot shows different frequency of interacting allele. (A - 0.05, B - 0.4, C - 0.6, D - 0.95).

These expressions can be substituted to expression (2) to obtain expected *NCP*. These formulas were validated by simulations and results are shown in Additional file 1, Figure S2. The power to detect *β*_*gF *_by direct test does not depend on effect of factor (*F*) as opposed to the homogeneity test. Figure 3 shows dependence of non-centrality parameter of variance homogeneity test on effect of factor for different frequencies of interacting allele *P*_*B *_= {0.05, 0.4, 0.6, 0.95} and different effects of interaction: the top curve on each plot shows results for interaction effect equals *β*_*gF *_= 1, the middle curve is for *β*_*gF *_= 0.5, and the bottom curve is for *β*_*gF *_= 0.1.

One can see that non-centrality parameter grows with increasing of interaction effect and minor allele frequency. The dependence is not monotonic and there are certain optimal effects of the factor $\beta_{F}^{opt}$, where the power to detect variance heterogeneity is maximum and minimum.

The plots for such dependence but for one degree of freedom tests are similar. They are shown in Additional file 1, Figures S3, S4 and S5.

It is of interest to note that *NCP *curves at complementary *P*_*B *_(say 0.05 and 0.95) may look like mirror images at first glance: however, this symmetry is not complete. Asymmetry between plots for complementary frequencies can be explained by taking into account that heterogeneity of variances for a case $P_{B}^{2}<<2P_{B}(1-P_{B})$, when genotype *BB *can be neglected, is determined mostly by:

$$\frac{\sigma_{AB}^{2}-\sigma_{AA}^{2}}{\sigma_{AA}^{2}}=\frac{\beta_{gF}^{2}+2\beta_{gF}\beta_{F}}{\beta_{F}^{2}+\frac{\sigma_{\epsilon }^{2}}{\sigma_{F}^{2}}}$$

whereas in an opposite case, when genotype *AA *is neglected, heterogeneity of variances is determined by

$$\frac{\sigma_{BB}^{2}-\sigma_{AB}^{2}}{\sigma_{AB}^{2}}=\frac{3\beta_{gF}^{2}+2\beta_{gF}\beta_{F}}{\beta_{gF}^{2}+2\beta_{gF}\beta_{F}+\beta_{F}^{2}+\frac{\sigma_{\epsilon }^{2}}{\sigma_{F}^{2}}}$$

The optimal effect of factor in the first case is given by

(6) $$\beta_{F,AAvsAB}^{opt}=\frac{-\beta_{gF}\pm \sqrt{\beta_{gF}^{2}+4\frac{\sigma_{\epsilon }^{2}}{\sigma_{F}^{2}}}}{2}$$

Similarly, in second case,

(7) $$\beta_{F,ABvsBB}^{opt}=\frac{-3\beta_{gF}\pm \sqrt{\beta_{gF}^{2}+4\frac{\sigma_{\epsilon }^{2}}{\sigma_{F}^{2}}}}{2}$$

**Figure 4:**
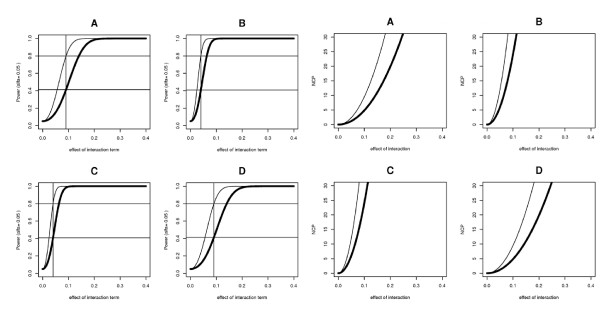
**Dependence of power to detect interaction (left plot) with threshold corresponding to *α *= 0.05 and non-centrality parameter (right plot) on effect of interaction**. Thin curve on each subplot corresponds to direct test, bold curve corresponds to upper limit of variance homogeneity test. Each subplot corresponds to different frequency of interacting allele (A - 0.05, B - 0.4, C - 0.6, D - 0.95).

Figure 4 shows analytical curves of dependence of power to detect interaction on effect of interaction for direct and variance homogeneity tests. Light curves present power of direct test, darker curve - upper limit of power of variance homogeneity test.

Such a dependence but for threshold corresponding to *α *= 5·10^-8 ^and *α *= 0.01 is shown in Additional file 1, Figure S6.

Table 1 presents power of variance homogeneity test under optimal effect of factor when power of direct test is 80%. Each column presents allele frequency of interacting allele (0.05, 0.4, 0.6, 0.95), and each row presents threshold *α *(0.05, 0.01, 5·10^-8^).

**Table 1:** Power of variance homogeneity test under optimal effect of factor when power of direct test is 80%.

	5%	40%	60%	95%
0.05	0.414	0.409	0.409	0.414
0.01	0.342	0.334	0.334	0.342
5·10^-8^	0.125	0.107	0.107	0.125

Each column presents allele frequency of interacting allele, each row presents threshold *α*.

## Performance of proposed method on real data

In order to measure the performance of the proposed method using clinical data, we applied Levene's variance homogeneity test on genome wide data for C-reactive protein (CRP), an inflammatory marker in the Rotterdam Study.

The Rotterdam Study (RS) [7] is a prospective cohort study that started in 1990 in Ommoord, a suburb of Rotterdam, and consists of 10,994 men and women aged 55 and over. The main objectives of the Rotterdam Study are to investigate prevalence, incidence and risk factors for cardiovascular, neurological, locomotor, and ophthalmologic diseases in the elderly. In the Rotterdam Study, genome-wide SNP genotyping was performed using Infinium II assay on the HumanHap550 Genotyping BeadChips (Illumina Inc., San Diego, CA, USA). In the present work, we used 5959 participants for whom genome wide and CRP data were available. Prior of applying the variance homogeneity test logarithmic transformation of CRP was performed. Genotypes from the selected SNP were tested separately. Additional file 1, figure S7 shows genome-wide log(p-value) plot and Q-Q plots respectively. Results show that no SNPs reached genome-wide significance level. The lowest *p*_*value *_= 4.77^-06 ^corresponded to SNP rs2399332 which is located on chromosome 3.

In the work of Guillaume Pare et al [6] Levene's test was applied to study CRP on a sample size of 21, 799 women, and results showed a significant SNP rs12753193 located on chromosome 1 showed the lowest *p*_*value *_= 1.6^-29^. We tested the same SNP in Rotterdam Study and found a *p*_*value *_of 0.011, with minor allele frequency of 0.385 for the risk-allele "G". The trait variances (and sample size) for genotypes *AA *(*n *= 2098), *AG *(*n *= 2643), and *GG *(*n *= 808) were 1.04, 1.10, and 1.18 respectively. Similarly to the work [6] genotype *GG *has the largest variance. From this result, we validated the genetic variant rs12753193 in the Rotterdam Study population.

## Discussion

Assuming that a genotype interacts with some factor in determination of a trait's value, it is expected that the trait's variance is increased in the group of subjects having this genotype. Thus, test of heterogeneity of variances can be proposed as a test to screen for potentially interacting SNPs. In this work, we evaluated type I error and power of variance heterogeneity analysis in respect to the detection of potentially interacting SNPs under the scenario when an interaction variable is unknown.

Three different tests of variance homogeneity were chosen in order to investigate their type I error performance. They are Bartlett's, Bartlett's with prior rank-transformation to normality of a trait and Levene's (Brown-Forsythe) tests. Not surprisingly, our results were in agreement with what is known from standard statistical theory [8-11]: it is known that for Bartlett's departure of the distribution of analyzed trait from normality (e.g. skewness or heavy tails) lead to increased type I error and Levene's test has better performance under these conditions. Interestingly, we have found that Bartlett's test has increased type I error even when the distribution of the trait is forced to be perfectly normal by application of rank transformation to normality in the case when the original pre-transformed distribution was non-normal, and direct effect of the SNP is present. These results, which may seem surprising at first, may be easily explained: three non-normal distributions with the same variance but different means after transformation translate to still not normal distributions with different variances. An illustrative example is provided in Additional file 1, Figure S1.

We showed that even if a large interaction effect is present, the power of the "screening" variance heterogeneity test depends strongly on the main effect of the interacting factor and may be quite limited. This results may at first seem surprising and contra-intuitive. To help better understanding of this phenomenon, here we provide a simple example of situation when there is an interaction effect, but the variances for all genotypes are equal, thus the variance test has no power. Consider binary factor **F **∈ {-1, 1} with effect on the trait - in accordance to our previous notation - equal to *β*_*F *_, and frequency of "1" denoted as *f *(thus frequency of "-1" is 1 - *f*). Let genotype in question to be "dominant" and coded as *g *∈ {0, 1, 1} for genotypes {*AA*, *AB*, *BB*}, respectively. Let mean *μ *= 0; for simplicity, at first, let us assume that the main effect of genotype is *β*_*g *_= 0. Let us denote the effect of genotype by factor interaction as *β*_*gF *_. Let the residual variance is $\sigma_{\epsilon }^{2}$. In this case, the conditional expectations of the trait for the genotype "0" are *E*(*y*|*g *= 0, **F **= -1) = -*β*_*F *_(when the value of factor is -1) and *E*(*y*|*g *= 0, **F **= 1) = *β*_*F *_. For genotype "1", the expectations are *E*(*y*|*g *= 1, **F **= -1) = -*β*_*F *_- *β*_*gF *_and *E*(*y*|*g *= 1, **F **= 1) = *β*_*F *_+ *β*_*gF *_. It is easy to see that the conditional variance of the trait in genotype *g *= 0 is simply $Var(y|g=0)=\sigma_{\epsilon }^{2}+4\beta_{F}^{2}f(1-f)$, while the variance of the trait in other genotype is $Var(y|g=1)=\sigma_{\epsilon }^{2}+4{\left(\beta_{F}+\beta_{gF}\right)}^{2}f(1-f)$. The conditional variances of the two genotypes are equal when either of two conditions is met: *β*_*gF *_= 0 (absence of interaction) or *β*_*F *_= -*β*_*gF*_/2. Taking a simple example with *f *= 1/2 it is straightforward to see how the variance could be the same while interaction effect is present. Interestingly, if *f *≠ 1/2 and *β*_*F *_= -*β*_*gF*_/2, the conditional variances *Var*(*y*|*g *= 0) = *Var*(*y*|*g *= 1), but conditional expectations *E*(*y*|*g *= 0) ≠ *E*(*y*|*g *= 1), so the interaction will translate into marginal SNP effect in the absence of the main effect (we assumed that *β*_*g *_= 0). As *β*_*F *_deviates from -*β*_*gF*_/2 in any direction, the conditional variance *Var*(*y*|*g *= 1) will increase while *Var*(*y*|*g *= 0) will stay the same. With |*β*_*F *_| → ∞, *Var*(*y*|*g *= 1) → *Var*(*y*|*g *= 0). This explains the non-monotonic, M-shaped dependency of the non-centrality parameter of variance test on the main effect of the interaction variable demonstrated in Figure 2.

While in this work we consider a model assuming a SNP having additive effect and following Hardy-Weinberg distribution and an interaction factor following normal distribution, the same principal result - non-monotonic dependence of the power of variance test on the main effect of interacting variable - should hold for other models and other types of interacting factor (e.g. binary, as we show above, or three-level, such as other SNPs); also, a deviation from HWE will not affect our major conclusions.

Our analysis of power was performed using Bartlett's test. Bartlett's has highest power in case of normally distributed trait, but is not robust to non-normality in trait distribution. Levene's test has better performance under deviations from normality, but has lower power compared to Bartlett's test. Therefore our principal findings will not change whether Bartlett's or Levene's test is used: particular figures provided estimate maximal power, but the relation of the power to the underlying model parameters will be the same for both tests.

We considered testing for heterogeneity of variances as a screening tool for potentially interacting SNPs in the context of population-based design. It has been proposed that this testing can be more effectively done in the context of monozygotic twins or migrant studies [4]. While these designs may indeed be more powerful compared to population-based design, the same relation between power of variance heterogeneity test and the underlying model parameters is to be expected in these designs as well.

Thus, for a wide range of designs, models and test used, we can conclude that that absence of significant heterogeneity of variances can not be interpreted as absence of strong interaction because the power of the variance test depends much on the main effect of the (unobserved) interacting factor.

It is interesting to consider whether presence of significant variance heterogeneity tells us that a SNP indeed interacts with some factor. First of all, variance heterogeneity will be detected for a SNP having main effect when the distribution of the trait is heteroscedastic, i.e. the variance increases with the mean - a situation rather common in biology. This suggests that prior test for heteroscedasity should be performed before running variance heterogeneity as an "interaction screening" test. Another - biological - possibility is that a genotype indeed affects the variance of the trait without any specific interaction. We can speculate that there may be genotypes which affect the stability of development or homeostasis, leading to wider trait's variance.

Detection of a variance homogeneity for a given SNP does not necessary indicate that a single factor is interacting with a studied SNP. Moreover, it can suggest the presence of a complex network with many other SNPs and factors involved. The variance heterogeneity test may be especially effective to detect such SNPs - in case of multiple interacting factors it is very unlikely that the cumulative effects of the interacting factor will fall into the point at which the power of the variance test is minimal.

Further dissection of the SNPs demonstrating strong heterogeneity of variances may be a challenging task, requiring the search of the interactors through phenomic screening. Straightforward testing whether the identified interactor does explain heterogeneity of variances can be easily performed by using the variance homogeneity test on the residuals from the regression involving identified factor.

A number of genetic interaction models may lead to variance heterogeneity. These are straightforward interaction models as discussed above, when an environmental of other genetic factor changes the expectation of the trait value in the concert with the SNP studied. Other interesting model, leading to specific increase of the variance of the heterozygous genotype, is parent-of-origin model, when the expectation of the trait in heterozygous individuals (*AB*) depends on whether allele *A *was transmitted from father or from mother.

We showed that when one interacting factor is considered, the power of direct test, exploiting the knowledge of the interacting factor, is always greater then the power of the variance heterogeneity test. An interesting scenario in which the power of variance heterogeneity test may be greater than the power of direct test occurs when multiple interacting factors induce variance heterogeneity, in which case the power of identification any single of them (or all together) may be - due to small effects associated with particular interacting factor and with increased number of degrees of freedom - lower then the power of variance heterogeneity test.

In present GWAS, association between a SNP and a trait is studied by detecting difference between mean values of the genotypes for a given SNP. We conclude that screening for differences in variances is a promising approach as a number of biologically interesting models may lead to the heterogeneity of variances. However, it should be clearly considered that absence of variance heterogeneity for a SNP can not be interpreted as absence of involvement of the SNP into interactions network, while the presence of significant heterogeneity may be explained not only by plain interaction with some factor, but also by other biological mechanisms and statistical artifacts.

## Conclusion

The method have been proposed for genome wide search of interaction between a SNP and a factor. The method is based on testing of variance homogeneity of a trait distributions in genotypes in which no knowledge of a factor is present. We have investigated type I error and power of three variance homogeneity tests (i.e. Bartlett's, Bartlett's with prior rank transformation of a trait to normality, and Levene's). Under variation of model parameters and distribution of residual errors only Levene's test kept acceptable type I error. We have obtained an analytical expression for power to detect interaction of direct test and variance homogeneity test. We also showed that the power of variance homogeneity test has lower power comparing to direct test under any model parameters when a single interacting variable is considered. As opposed to direct test, power of variance homogeneity test depends on the main effect of a factor. This dependency is non monotonic and for a given factor effect and it has its own maximums and minimums. By replicating the results of previous study [6], we demonstrate that application of the method can lead to biologically interesting, reproducible results.

## Authors' contributions

MS planned and carried out the simulation study, obtained analytical expressions, wrote the manuscript, and analyzed the data. AD, and JW provided data for the real example. CvD participated in planning and discussion of the study. YA planned simulation study, obtained analytical expressions and wrote the manuscript. All authors read and approved the final manuscript.

## Additional Files

Additional file 1 presents supplementary figures. Additional files 2, 3 and 4 present type I error for three investigated variance homogeneity tests (Bartlett's, Bartlett's with prior rank transformation of a trait to normality, and Levene's tests) and for seven types of distribution of residual errors (normally distributed, three types of *t *distribution and three types of *χ*^2 ^distribution with different degrees of freedom). The data is presented for a model which determine a trait where is effect of SNP presented which explains 0%, 1%, and 5% of total trait's variance.

## Supplementary Material

Additional file 1 **Supplementary figures**. The file contains the following figures: Figure S1: Distribution of a trait for each genotypic groups and for all groups together before transformation to normality of a trait and after transformation. Figure S2: Dependence of power on interaction effect for direct test and different variance homogeneity tests. Figure S3: Dependence of non-centrality parameter of variance homogeneity test on effect of a factor for a case when group AA is tested against AB and BB. Figure S4: Dependence of non-centrality parameter of variance homogeneity test on effect of a factor for a case when group AB is tested against AA and BB. Figure S5: Dependence of non-centrality parameter of variance homogeneity test on effect of a factor for a case when group BB is tested against AA and AB. Figure S6: Dependence of power of variance homogeneity test on interaction effect for threshold α corresponding to 5·10^-8 ^and 0.01. Figure S7: Genome-wide -*log*10(*p*_*value*_) and Q-Q plot for Levene's variance homogeneity test applied for the Rotterdam Study. Click here for file

Additional file 2 **Type I error for a case when all three genotypes are tested against each other**. Type I error for variance homogeneity tests when there is effect of SNP which explains 0%, 1%, and 5% of total trait's variance for different frequency of interacting allele (5%, 10%, 25% and 50%) and for different distribution of residual error (normal, three types of t and chi square distribution). Click here for file

Additional file 3 **Type I error for a case when genotype AA is tested against AB and BB**. Type I error for 1df variance homogeneity tests when AA is tested against AB and BB when there is effect of SNP which explains 0%, 1%, and 5% of total trait's variance for different frequency of interacting allele (5%, 10%, 25% and 50%) and for different distribution of residual error (normal, three types of t and chi square distribution ). Click here for file

Additional file 4 **Type I error for a case when genotype AB is tested against AA and BB**. Type I error for 1df variance homogeneity tests when AB is tested against AA and BB when there is effect of SNP which explains 0%, 1%, and 5% of total trait's variance for different frequency of interacting allele (5%, 10%, 25% and 50%) and for different distribution of residual error (normal, three types of t and chi square distribution ). Click here for file

Additional file 5 **Type I error for a case when genotype BB is tested against AB and AA**. Type I error for 1df variance homogeneity tests when BB is tested against AA and AB when there is effect of SNP which explains 0%, 1%, and 5% of total trait's variance for different frequency of interacting allele (5%, 10%, 25% and 50%) and for different distribution of residual error (normal, three types of t and chi square distribution ). Click here for file

## References

[B1] Hindorff LA Sethupathy P Junkins HA Ramos EM Mehta JP Collins FS Manolio TA Potential etiologic and functional implications of genome-wide association loci for human diseases and traits Proc Natl Acad Sci USA 2009 106 23 9362 9367 10.1073/pnas.0903103106 19474294 PMC2687147

[B2] Maher B Personal genomes: The case of the missing heritability Nature 2008 456 7218 18 21 10.1038/456018a 18987709

[B3] Aulchenko YS Struchalin MV Belonogova NM Axenovich TI Weedon MN Hofman A Uitterlinden AG Kayser M Oostra BA van Duijn CM Janssens ACJW Borodin PM Predicting human height by Victorian and genomic methods Eur J Hum Genet 2009 17 8 1070 1075 10.1038/ejhg.2009.5 19223933 PMC2986552

[B4] Manolio TA Collins FS Cox NJ Goldstein DB Hindorff LA Hunter DJ McCarthy MI Ramos EM Cardon LR Chakravarti A Cho JH Guttmacher AE Kong A Kruglyak L Mardis E Rotimi CN Slatkin M Valle D Whittemore AS Boehnke M Clark AG Eichler EE Gibson G Haines JL Mackay TFC McCarroll SA Visscher PM Finding the missing heritability of complex diseases Nature 2009 461 7265 747 753 10.1038/nature08494 19812666 PMC2831613

[B5] Li Y Willer C Sanna S Abecasis G Genotype imputation Annu Rev Genomics Hum Genet 2009 10 387 406 10.1146/annurev.genom.9.081307.164242 19715440 PMC2925172

[B6] Pare G Cook NR Ridker PM Chasman DI On the use of variance per genotype as a tool to identify quantitative trait interaction effects: a report from the Women's Genome Health Study PLoS Genet 2010 6 6 e1000981 10.1371/journal.pgen.1000981 20585554 PMC2887471

[B7] Hofman A Breteler MMB van Duijn CM Janssen HLA Krestin GP Kuipers EJ Stricker BHC Tiemeier H Uitterlinden A Vingerling JR Witteman JCM The Rotterdam Study: 2010 objectives and design update Eur J Epidemiol 2009 24 9 553 572 10.1007/s10654-009-9386-z 19728115 PMC2744826

[B8] Levene H Robust tests for equality of variances 1960 Stanford University Press 278 292

[B9] Brown MB Forsythe AB Robust Tests for Equality of Variances Journal of the American Statistical 1974

[B10] Bartlett MS Properties of sufficiency and statistical tests Proceedings of the Royal Statistical Society Series

[B11] Snedecor GW Cochran WG Statistical Methods 1989 Iowa State University Press

