# Supplementary material for: Variance heterogeneity analysis for detection of potentially interacting genetic loci: method and its limitations
Source: BMC Genet. 2010 Oct 13;11:92. doi: 10.1186/1471-2156-11-92 (PMC2973850; doi:10.1186/1471-2156-11-92)
Supplement: Additional file 1 — Supplementary figures. The file contains the following figures: Figure S1: Distribution of a trait for each genotypic groups and for all groups together before transformation to normality of a trait and after transformation. Figure S2: Dependence of power on interaction effect for direct test and different variance homogeneity tests. Figure S3: Dependence of non-centrality parameter of variance homogeneity test on effect of a factor for a case when group AA is tested against AB and BB. Figure S4: Dependence of non-centrality parameter of variance homogeneity test on effect of a factor for a case when group AB is tested against AA and BB. Figure S5: Dependence of non-centrality parameter of variance homogeneity test on effect of a factor for a case when group BB is tested against AA and AB. Figure S6: Dependence of power of variance homogeneity test on interaction effect for threshold α corresponding to 5·10-8 and 0.01. Figure S7: Genome-wide -log10(pvalue) and Q-Q plot for Levene's variance homogeneity test applied for the Rotterdam Study. [file 1471-2156-11-92-S1.PDF]

Supplementary

It was shown in the results of type I error of investigated variance homogeneity tests that in a case of SNP effect presence rank transformation to normality of a trait which follows non-normal distribution results to perfectly normally distributed trait whereas distribution of each genotypic groups becomes distorted. Figure 1 explains such a deformation.

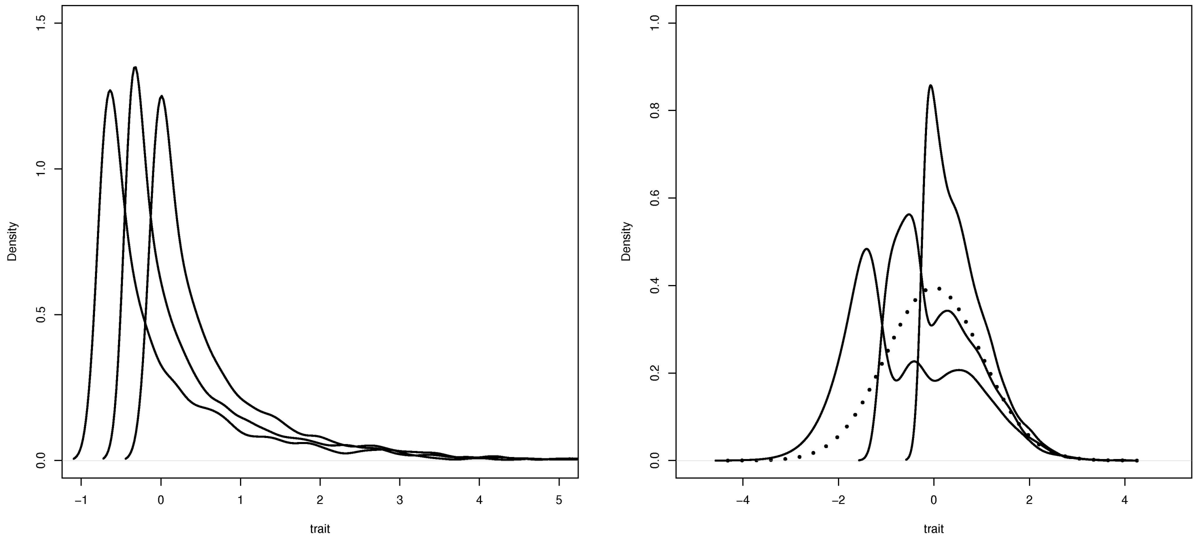

Figure 1: **Distribution of a trait for each genotypic groups (bold curves) and for all groups together (dotted curve) before transformation to normality of a trait (left) and after transformation (right).**

Analytical expressions for variances of trait's distribution in each genotypic group were obtained in this work. They can be used to obtain dependence of non-centrality parameter (and therefore power) on model parameters. To validate these analytical results simulations were done. Figure 2 shows analytical curves and simulated points for dependence of power on interaction effect for direct test and variance homogeneity tests.

Figures 3, 4, and 5 shows dependence of non-centrality parameter of variance homogeneity test on effect of a factor for a cases of one degree of freedom tests: when AA is tested against AB and BB, AB against AA and BB, BB against AA and AB.

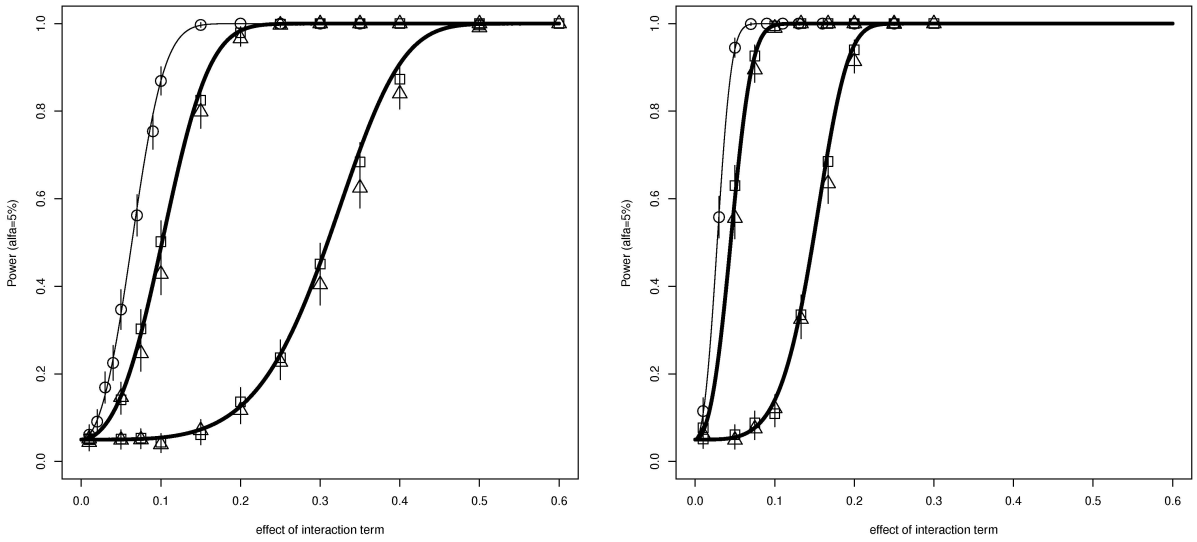

Figure 2: **Dependence of power on interaction effect for direct test and different variance homogeneity tests.** The thin curves on the left in each subplot corresponds to analytical expression of power of direct test. The two bold curves in each subplot corresponds to analytical expression of variance homogeneity test. The left bold curve is for a case of absence effect of a factor, the right bold curve is for effect of a factor one. The points correspond to simulations for direct (circles), Bartlett's (squares) and Levene's (triangles) tests. The left plot is for the case when frequency of interacting allele is 5% and no SNP effect. The right plot is for interacting allele frequency 50% and snp effect 0.3.

Figures 6 show dependence of power of variance homogeneity test with two degrees of freedom on interaction effect for threshold α corresponding to $5 \cdot 10^{-8}$ and 0.01.

Figures 7 show Genome-wide log(p-value) and Q-Q plot for Levene's variance homogeneity test applied for the Rotterdam Study. Q-Q plot presents only those SNPs which have three genotypes. As one can see there is no SNPs reached genome-wide significance level.

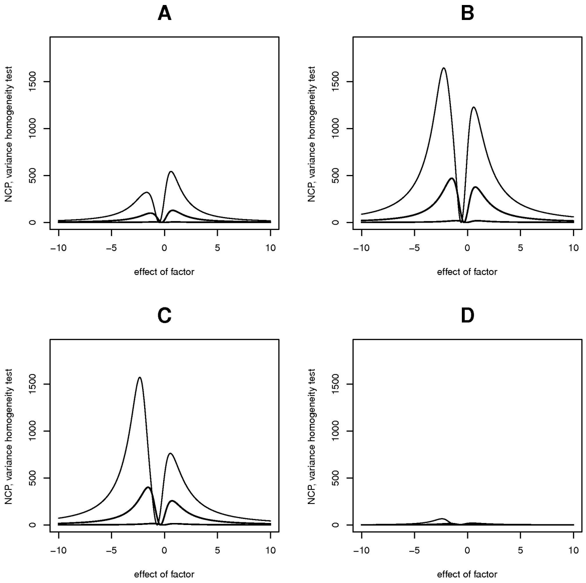

Figure 3: **Dependence of non-centrality parameter of variance homogeneity test on effect of a factor for a case when group AA is tested against AB and BB.** The top curve on each plot shows results for interaction effect equals $\beta_{gF} = 1$, the middle curve is for $\beta_{gF} = 0.5$, and the bottom curve is for $\beta_{gF} = 0.1$. Each subplot shows different frequency of interacting allele. (A – 0.05, B – 0.4, C – 0.6, D – 0.95).

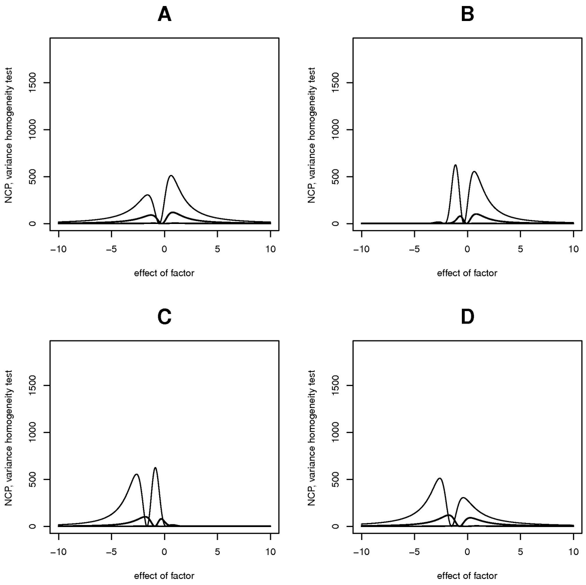

Figure 4: **Dependence of non-centrality parameter of variance homogeneity test on effect of a factor for a case when group AB is tested against AA and BB.** The top curve on each plot shows results for interaction effect equals $\beta_{gF} = 1$, the middle curve is for $\beta_{gF} = 0.5$, and the bottom curve is for $\beta_{gF} = 0.1$. Each subplot shows different frequency of interacting allele. (A – 0.05, B – 0.4, C – 0.6, D – 0.95).

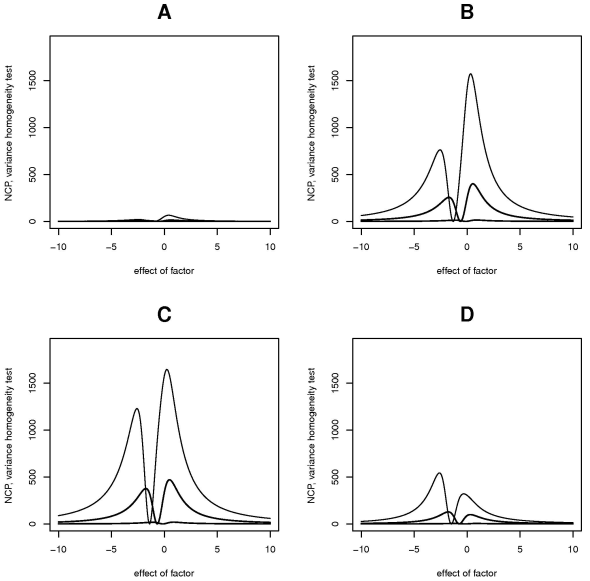

Figure 5: **Dependence of non-centrality parameter of variance homogeneity test on effect of a factor for a case when group BB is tested against AA and AB.** The top curve on each plot shows results for interaction effect equals $\beta_{gF} = 1$, the middle curve is for $\beta_{gF} = 0.5$, and the bottom curve is for $\beta_{gF} = 0.1$. Each subplot shows different frequency of interacting allele. (A – 0.05, B – 0.4, C – 0.6, D – 0.95).

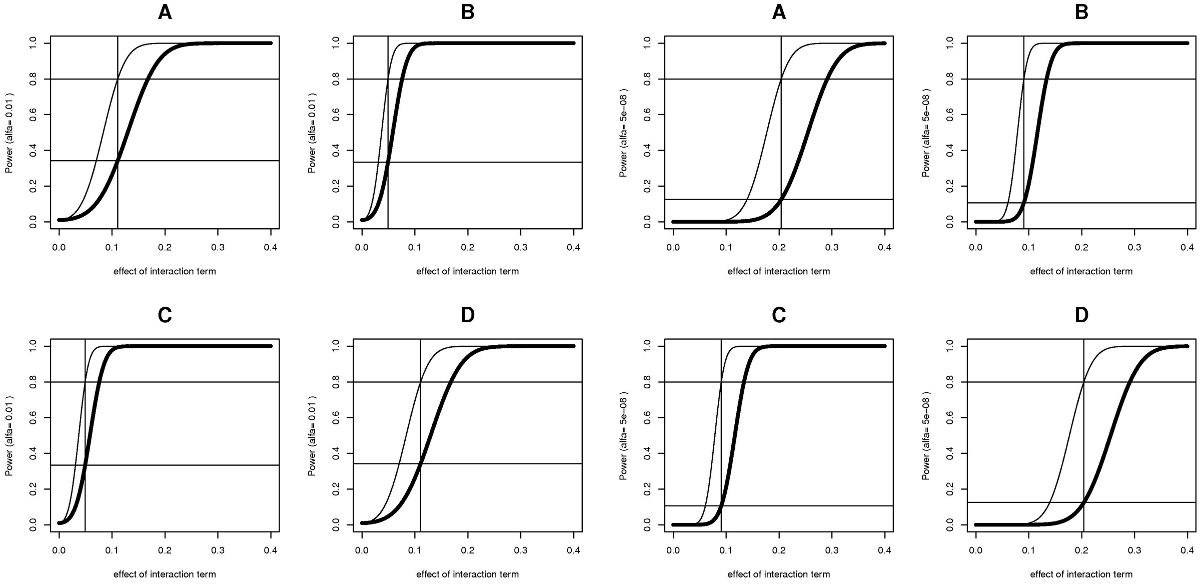

Figure 6: **Dependence of power of variance homogeneity test on interaction effect for threshold α corresponding to $5 \cdot 10^{-8}$ (left four plots) and 0.01 (right four plots).** Thin curve in each subplot corresponds to direct test, bold curve corresponds to upper limit of variance homogeneity test. Each subplot shows different frequency of interacting allele (A – 5%, B – 40%, C – 60%, D – 95%)

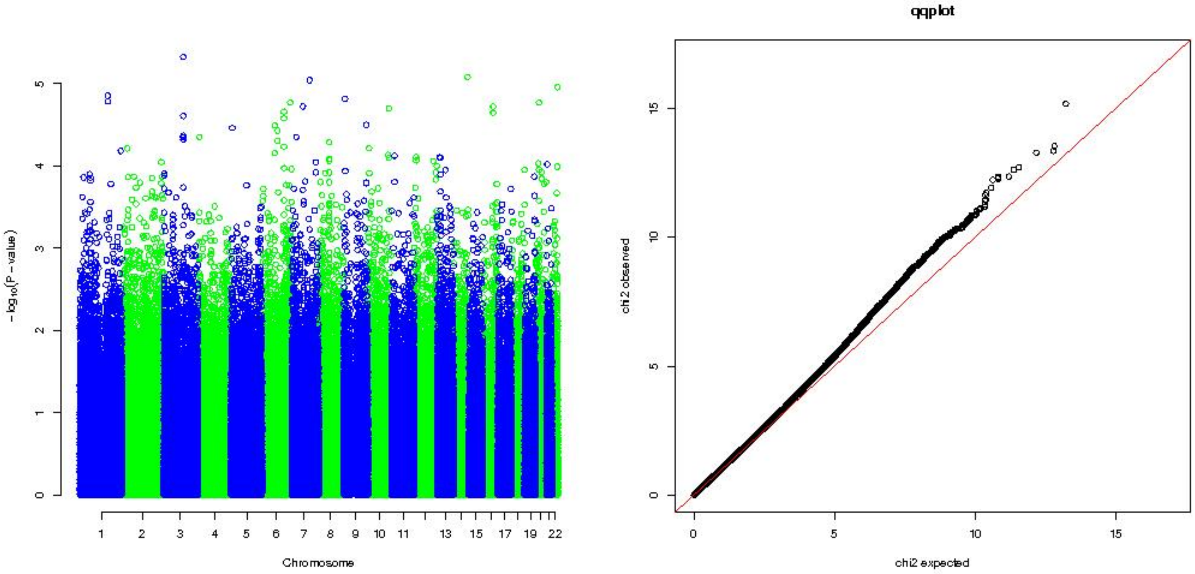

Figure 7: **Genome-wide log(p-value) and Q-Q plot for Levene's variance homogeneity test applied for the Rotterdam Study.**
