# Supplementary material for: Variance heterogeneity analysis for detection of potentially interacting genetic loci: method and its limitations
Source: BMC Genet. 2010 Oct 13;11:92. doi: 10.1186/1471-2156-11-92 (PMC2973850; doi:10.1186/1471-2156-11-92)
Supplement: Additional file 2 — Type I error for a case when all three genotypes are tested against each other. Type I error for variance homogeneity tests when there is effect of SNP which explains 0%, 1%, and 5% of total trait's variance for different frequency of interacting allele (5%, 10%, 25% and 50%) and for different distribution of residual error (normal, three types of t and chi square distribution). [file 1471-2156-11-92-S2.PDF]

Type I error for variance homogeneity tests when there is effect of SNP which explains 0%, 1%, and 5% of total trait's variance for different frequency of interacting allele (5%, 10%, 25% and 50%) and for different distribution of residual error (normal, three types of t and chi square distribution)

Table S1

Type I error for a case when there is no SNP

allele frequency 5%			
	**bartlett's**	**rank bartlett's**	**levene's**
**normal**	0.052+-0.002	0.051+-0.002	0.052+-0.002
**t, df=10**	0.12+-0.003	0.047+-0.002	0.045+-0.002
**t, df=5**	0.312+-0.005	0.05+-0.002	0.046+-0.002
**t, df=2**	0.962+-0.002	0.051+-0.002	0.051+-0.002
**chisq, df=15**	0.107+-0.003	0.046+-0.002	0.045+-0.002
**chisq, df=5**	0.225+-0.004	0.047+-0.002	0.044+-0.002
**chisq, df=1**	0.62+-0.005	0.05+-0.002	0.05+-0.002

allele frequency 10%			
	**bartlett's**	**rank bartlett's**	**levene's**
**normal**	0.048+-0.002	0.048+-0.002	0.049+-0.002
**t, df=10**	0.128+-0.003	0.05+-0.002	0.051+-0.002
**t, df=5**	0.354+-0.005	0.048+-0.002	0.048+-0.002
**t, df=2**	0.972+-0.002	0.05+-0.002	0.05+-0.002
**chisq, df=15**	0.116+-0.003	0.049+-0.002	0.049+-0.002
**chisq, df=5**	0.255+-0.004	0.049+-0.002	0.051+-0.002
**chisq, df=1**	0.634+-0.005	0.051+-0.002	0.044+-0.002

allele frequency 25%			
	**bartlett's**	**rank bartlett's**	**levene's**
**normal**	0.052+-0.002	0.052+-0.002	0.052+-0.002
**t, df=10**	0.138+-0.003	0.053+-0.002	0.052+-0.002
**t, df=5**	0.393+-0.005	0.05+-0.002	0.05+-0.002
**t, df=2**	0.982+-0.001	0.049+-0.002	0.052+-0.002
**chisq, df=15**	0.117+-0.003	0.051+-0.002	0.051+-0.002
**chisq, df=5**	0.255+-0.004	0.047+-0.002	0.049+-0.002
**chisq, df=1**	0.65+-0.005	0.052+-0.002	0.054+-0.002

allele frequency 50%			
	**bartlett's**	**rank bartlett's**	**levene's**
**normal**	0.05+-0.002	0.051+-0.002	0.051+-0.002
**t, df=10**	0.131+-0.003	0.047+-0.002	0.049+-0.002
**t, df=5**	0.405+-0.005	0.049+-0.002	0.049+-0.002
**t, df=2**	0.985+-0.001	0.05+-0.002	0.045+-0.002
**chisq, df=15**	0.116+-0.003	0.05+-0.002	0.049+-0.002
**chisq, df=5**	0.244+-0.004	0.048+-0.002	0.05+-0.002
**chisq, df=1**	0.646+-0.005	0.046+-0.002	0.051+-0.002

Table S2

Type I error for a case when there is SNP effect which expalins 1% of total trait's variance

allele frequency 5%			
	**bartlett's**	**rank bartlett's**	**levene's**
**normal**	0.047+-0.002	0.046+-0.002	0.045+-0.002
**t, df=10**	0.125+-0.003	0.061+-0.002	0.049+-0.002
**t, df=5**	0.318+-0.005	0.086+-0.003	0.053+-0.002
**t, df=2**	0.956+-0.002	0.555+-0.005	0.046+-0.002
**chisq, df=15**	0.109+-0.003	0.919+-0.003	0.044+-0.002
**chisq, df=5**	0.231+-0.004	1+-0	0.046+-0.002
**chisq, df=1**	0.615+-0.005	1+-0	0.046+-0.002

allele frequency 10%			
	**bartlett's**	**rank bartlett's**	**levene's**
**normal**	0.052+-0.002	0.049+-0.002	0.052+-0.002
**t, df=10**	0.135+-0.003	0.054+-0.002	0.049+-0.002
**t, df=5**	0.351+-0.005	0.063+-0.002	0.046+-0.002
**t, df=2**	0.977+-0.001	0.358+-0.005	0.049+-0.002
**chisq, df=15**	0.11+-0.003	0.932+-0.003	0.05+-0.002
**chisq, df=5**	0.248+-0.004	1+-0	0.044+-0.002
**chisq, df=1**	0.627+-0.005	1+-0	0.048+-0.002

allele frequency 25%			
	**bartlett's**	**rank bartlett's**	**levene's**
**normal**	0.05+-0.002	0.049+-0.002	0.05+-0.002
**t, df=10**	0.136+-0.003	0.053+-0.002	0.053+-0.002
**t, df=5**	0.395+-0.005	0.057+-0.002	0.051+-0.002
**t, df=2**	0.984+-0.001	0.192+-0.004	0.053+-0.002
**chisq, df=15**	0.12+-0.003	0.945+-0.002	0.051+-0.002
**chisq, df=5**	0.255+-0.004	1+-0	0.048+-0.002
**chisq, df=1**	0.651+-0.005	1+-0	0.049+-0.002

allele frequency 50%			
	**bartlett's**	**rank bartlett's**	**levene's**
**normal**	0.051+-0.002	0.048+-0.002	0.052+-0.002
**t, df=10**	0.131+-0.003	0.048+-0.002	0.046+-0.002
**t, df=5**	0.409+-0.005	0.055+-0.002	0.055+-0.002
**t, df=2**	0.985+-0.001	0.138+-0.003	0.046+-0.002
**chisq, df=15**	0.113+-0.003	0.951+-0.002	0.047+-0.002
**chisq, df=5**	0.254+-0.004	1+-0	0.05+-0.002
**chisq, df=1**	0.652+-0.005	1+-0	0.049+-0.002

Table S3

Type I error for a case when there is SNP effect which expalins 5% of total trait's variance

allele frequency 5%			
	bartlett's	rank bartlett's	levene's
normal	0.051+-0.002	0.05+-0.002	0.049+-0.002
t, df=10	0.127+-0.003	0.25+-0.004	0.052+-0.002
t, df=5	0.313+-0.005	0.531+-0.005	0.047+-0.002
t, df=2	0.96+-0.002	0.996+-0.001	0.051+-0.002
chisq, df=15	0.107+-0.003	1+-0	0.048+-0.002
chisq, df=5	0.231+-0.004	1+-0	0.046+-0.002
chisq, df=1	0.623+-0.005	1+-0	0.047+-0.002

allele frequency 10%			
	bartlett's	rank bartlett's	levene's
normal	0.051+-0.002	0.042+-0.002	0.052+-0.002
t, df=10	0.135+-0.003	0.137+-0.003	0.048+-0.002
t, df=5	0.359+-0.005	0.3+-0.005	0.048+-0.002
t, df=2	0.974+-0.002	0.968+-0.002	0.049+-0.002
chisq, df=15	0.117+-0.003	1+-0	0.048+-0.002
chisq, df=5	0.251+-0.004	1+-0	0.052+-0.002
chisq, df=1	0.637+-0.005	1+-0	0.048+-0.002

allele frequency 25%			
	bartlett's	rank bartlett's	levene's
normal	0.051+-0.002	0.043+-0.002	0.047+-0.002
t, df=10	0.132+-0.003	0.078+-0.003	0.052+-0.002
t, df=5	0.39+-0.005	0.135+-0.003	0.048+-0.002
t, df=2	0.982+-0.001	0.729+-0.004	0.051+-0.002
chisq, df=15	0.117+-0.003	1+-0	0.049+-0.002
chisq, df=5	0.253+-0.004	1+-0	0.052+-0.002
chisq, df=1	0.649+-0.005	1+-0	0.052+-0.002

allele frequency 50%			
	bartlett's	rank bartlett's	levene's
normal	0.049+-0.002	0.04+-0.002	0.051+-0.002
t, df=10	0.137+-0.003	0.06+-0.002	0.049+-0.002
t, df=5	0.403+-0.005	0.102+-0.003	0.052+-0.002
t, df=2	0.986+-0.001	0.54+-0.005	0.043+-0.002
chisq, df=15	0.119+-0.003	1+-0	0.051+-0.002
chisq, df=5	0.253+-0.004	1+-0	0.046+-0.002
chisq, df=1	0.645+-0.005	1+-0	0.05+-0.002
