# Supplementary material for: Variance heterogeneity analysis for detection of potentially interacting genetic loci: method and its limitations
Source: BMC Genet. 2010 Oct 13;11:92. doi: 10.1186/1471-2156-11-92 (PMC2973850; doi:10.1186/1471-2156-11-92)
Supplement: Additional file 3 — Type I error for a case when genotype AA is tested against AB and BB. Type I error for 1df variance homogeneity tests when AA is tested against AB and BB when there is effect of SNP which explains 0%, 1%, and 5% of total trait's variance for different frequency of interacting allele (5%, 10%, 25% and 50%) and for different distribution of residual error (normal, three types of t and chi square distribution ). [file 1471-2156-11-92-S3.PDF]

Type I error for 1df variance homogeneity tests when AA genotype is tested against AB and BB in a case there is effect of SNP which explains 0%, 1%, and 5% of total trait's variance for different frequency of interacting allele (5%, 10%, 25% and 50%) and for different distribution of residual error (normal, three types of t and chi square distribution).

Table S1

Type I error for a case when there is no SNP effect

allele frequency 5%			
	**bartlett's**	**rank bartlett's**	**levene's**
**normal**	0.05+-0.002	0.049+-0.002	0.052+-0.002
**t, df=10**	0.109+-0.003	0.048+-0.002	0.048+-0.002
**t, df=5**	0.264+-0.004	0.05+-0.002	0.048+-0.002
**t, df=2**	0.895+-0.003	0.057+-0.002	0.057+-0.002
**chisq, df=15**	0.096+-0.003	0.082+-0.003	0.049+-0.002
**chisq, df=5**	0.187+-0.004	0.232+-0.004	0.049+-0.002
**chisq, df=1**	0.452+-0.005	0.95+-0.002	0.05+-0.002

allele frequency 10%			
	**bartlett's**	**rank bartlett's**	**levene's**
**normal**	0.051+-0.002	0.05+-0.002	0.051+-0.002
**t, df=10**	0.11+-0.003	0.05+-0.002	0.05+-0.002
**t, df=5**	0.277+-0.004	0.052+-0.002	0.05+-0.002
**t, df=2**	0.899+-0.003	0.055+-0.002	0.055+-0.002
**chisq, df=15**	0.099+-0.003	0.085+-0.003	0.048+-0.002
**chisq, df=5**	0.186+-0.004	0.229+-0.004	0.05+-0.002
**chisq, df=1**	0.457+-0.005	0.94+-0.002	0.046+-0.002

allele frequency 25%			
	**bartlett's**	**rank bartlett's**	**levene's**
**normal**	0.05+-0.002	0.05+-0.002	0.051+-0.002
**t, df=10**	0.106+-0.003	0.048+-0.002	0.05+-0.002
**t, df=5**	0.282+-0.005	0.048+-0.002	0.047+-0.002
**t, df=2**	0.907+-0.003	0.055+-0.002	0.047+-0.002
**chisq, df=15**	0.095+-0.003	0.086+-0.003	0.051+-0.002
**chisq, df=5**	0.186+-0.004	0.229+-0.004	0.049+-0.002
**chisq, df=1**	0.469+-0.005	0.922+-0.003	0.052+-0.002

allele frequency 50%			
	**bartlett's**	**rank bartlett's**	**levene's**
**normal**	0.052+-0.002	0.051+-0.002	0.051+-0.002
**t, df=10**	0.111+-0.003	0.048+-0.002	0.051+-0.002
**t, df=5**	0.285+-0.005	0.05+-0.002	0.053+-0.002
**t, df=2**	0.901+-0.003	0.056+-0.002	0.05+-0.002
**chisq, df=15**	0.098+-0.003	0.086+-0.003	0.051+-0.002
**chisq, df=5**	0.189+-0.004	0.236+-0.004	0.049+-0.002
**chisq, df=1**	0.456+-0.005	0.905+-0.003	0.045+-0.002

Table S2

Type I error for a case when there is SNP effect which expalins 1% of total trait's variance

allele frequency 5%			
	bartlett's	rank bartlett's	levene's
normal	0.052+-0.002	0.052+-0.002	0.052+-0.002
t, df=10	0.105+-0.003	0.046+-0.002	0.047+-0.002
t, df=5	0.278+-0.004	0.053+-0.002	0.052+-0.002
t, df=2	0.89+-0.003	0.052+-0.002	0.058+-0.002
chisq, df=15	0.099+-0.003	0.087+-0.003	0.049+-0.002
chisq, df=5	0.186+-0.004	0.236+-0.004	0.051+-0.002
chisq, df=1	0.46+-0.005	0.948+-0.002	0.048+-0.002

allele frequency 10%			
	bartlett's	rank bartlett's	levene's
normal	0.053+-0.002	0.053+-0.002	0.05+-0.002
t, df=10	0.112+-0.003	0.049+-0.002	0.048+-0.002
t, df=5	0.281+-0.004	0.049+-0.002	0.049+-0.002
t, df=2	0.9+-0.003	0.054+-0.002	0.055+-0.002
chisq, df=15	0.092+-0.003	0.086+-0.003	0.051+-0.002
chisq, df=5	0.186+-0.004	0.231+-0.004	0.049+-0.002
chisq, df=1	0.468+-0.005	0.942+-0.002	0.053+-0.002

allele frequency 25%			
	bartlett's	rank bartlett's	levene's
normal	0.049+-0.002	0.049+-0.002	0.051+-0.002
t, df=10	0.108+-0.003	0.048+-0.002	0.048+-0.002
t, df=5	0.29+-0.005	0.049+-0.002	0.051+-0.002
t, df=2	0.904+-0.003	0.047+-0.002	0.043+-0.002
chisq, df=15	0.095+-0.003	0.083+-0.003	0.047+-0.002
chisq, df=5	0.185+-0.004	0.229+-0.004	0.052+-0.002
chisq, df=1	0.455+-0.005	0.92+-0.003	0.046+-0.002

allele frequency 50%			
	bartlett's	rank bartlett's	levene's
normal	0.05+-0.002	0.05+-0.002	0.05+-0.002
t, df=10	0.107+-0.003	0.048+-0.002	0.049+-0.002
t, df=5	0.294+-0.005	0.052+-0.002	0.052+-0.002
t, df=2	0.902+-0.003	0.052+-0.002	0.048+-0.002
chisq, df=15	0.1+-0.003	0.088+-0.003	0.054+-0.002
chisq, df=5	0.187+-0.004	0.227+-0.004	0.049+-0.002
chisq, df=1	0.447+-0.005	0.902+-0.003	0.048+-0.002

Table S3

Type I error for a case when there is SNP effect which expalins 5% of total trait's variance

allele frequency 5%			
	**bartlett's**	**rank bartlett's**	**levene's**
**normal**	0.051+-0.002	0.05+-0.002	0.051+-0.002
**t, df=10**	0.107+-0.003	0.05+-0.002	0.049+-0.002
**t, df=5**	0.274+-0.004	0.049+-0.002	0.048+-0.002
**t, df=2**	0.894+-0.003	0.051+-0.002	0.053+-0.002
**chisq, df=15**	0.099+-0.003	0.089+-0.003	0.05+-0.002
**chisq, df=5**	0.186+-0.004	0.234+-0.004	0.052+-0.002
**chisq, df=1**	0.46+-0.005	0.952+-0.002	0.051+-0.002

allele frequency 10%			
	**bartlett's**	**rank bartlett's**	**levene's**
**normal**	0.05+-0.002	0.05+-0.002	0.052+-0.002
**t, df=10**	0.11+-0.003	0.052+-0.002	0.051+-0.002
**t, df=5**	0.27+-0.004	0.049+-0.002	0.049+-0.002
**t, df=2**	0.903+-0.003	0.051+-0.002	0.053+-0.002
**chisq, df=15**	0.095+-0.003	0.083+-0.003	0.049+-0.002
**chisq, df=5**	0.183+-0.004	0.23+-0.004	0.048+-0.002
**chisq, df=1**	0.458+-0.005	0.94+-0.002	0.05+-0.002

allele frequency 25%			
	**bartlett's**	**rank bartlett's**	**levene's**
**normal**	0.05+-0.002	0.049+-0.002	0.048+-0.002
**t, df=10**	0.107+-0.003	0.048+-0.002	0.047+-0.002
**t, df=5**	0.288+-0.005	0.05+-0.002	0.05+-0.002
**t, df=2**	0.907+-0.003	0.051+-0.002	0.044+-0.002
**chisq, df=15**	0.095+-0.003	0.082+-0.003	0.047+-0.002
**chisq, df=5**	0.189+-0.004	0.24+-0.004	0.051+-0.002
**chisq, df=1**	0.455+-0.005	0.92+-0.003	0.048+-0.002

allele frequency 50%			
	**bartlett's**	**rank bartlett's**	**levene's**
**normal**	0.046+-0.002	0.047+-0.002	0.046+-0.002
**t, df=10**	0.105+-0.003	0.045+-0.002	0.045+-0.002
**t, df=5**	0.286+-0.005	0.054+-0.002	0.054+-0.002
**t, df=2**	0.906+-0.003	0.05+-0.002	0.044+-0.002
**chisq, df=15**	0.1+-0.003	0.089+-0.003	0.05+-0.002
**chisq, df=5**	0.19+-0.004	0.236+-0.004	0.052+-0.002
**chisq, df=1**	0.47+-0.005	0.905+-0.003	0.053+-0.002
