# Supplementary material for: Variance heterogeneity analysis for detection of potentially interacting genetic loci: method and its limitations
Source: BMC Genet. 2010 Oct 13;11:92. doi: 10.1186/1471-2156-11-92 (PMC2973850; doi:10.1186/1471-2156-11-92)
Supplement: Additional file 4 — Type I error for a case when genotype AB is tested against AA and BB. Type I error for 1df variance homogeneity tests when AB is tested against AA and BB when there is effect of SNP which explains 0%, 1%, and 5% of total trait's variance for different frequency of interacting allele (5%, 10%, 25% and 50%) and for different distribution of residual error (normal, three types of t and chi square distribution ). [file 1471-2156-11-92-S4.PDF]

Type I error for 1df variance homogeneity tests when AB genotype is tested against AA and BB in a case there is effect of SNP which explains 0%, 1%, and 5% of total trait's variance for different frequency of interacting allele (5%, 10%, 25% and 50%) and for different distribution of residual error (normal, three types of t and chi square distribution).

Table S1

Type I error for case when there is no SNP effect

B allele frequency 5%			
	**bartlett's**	**rank bartlett's**	**levene's**
**normal**	0.05+-0.002	0.049+-0.002	0.052+-0.002
**t, df=10**	0.109+-0.003	0.049+-0.002	0.048+-0.002
**t, df=5**	0.266+-0.004	0.05+-0.002	0.047+-0.002
**t, df=2**	0.896+-0.003	0.054+-0.002	0.054+-0.002
**chisq, df=15**	0.097+-0.003	0.084+-0.003	0.048+-0.002
**chisq, df=5**	0.188+-0.004	0.233+-0.004	0.049+-0.002
**chisq, df=1**	0.454+-0.005	0.976+-0.002	0.048+-0.002

allele frequency 10%			
	**bartlett's**	**rank bartlett's**	**levene's**
**normal**	0.048+-0.002	0.048+-0.002	0.05+-0.002
**t, df=10**	0.11+-0.003	0.049+-0.002	0.05+-0.002
**t, df=5**	0.274+-0.004	0.051+-0.002	0.05+-0.002
**t, df=2**	0.898+-0.003	0.055+-0.002	0.054+-0.002
**chisq, df=15**	0.099+-0.003	0.082+-0.003	0.047+-0.002
**chisq, df=5**	0.184+-0.004	0.229+-0.004	0.052+-0.002
**chisq, df=1**	0.454+-0.005	0.977+-0.001	0.045+-0.002

allele frequency 25%			
	**bartlett's**	**rank bartlett's**	**levene's**
**normal**	0.052+-0.002	0.051+-0.002	0.05+-0.002
**t, df=10**	0.108+-0.003	0.05+-0.002	0.049+-0.002
**t, df=5**	0.28+-0.004	0.052+-0.002	0.049+-0.002
**t, df=2**	0.905+-0.003	0.053+-0.002	0.046+-0.002
**chisq, df=15**	0.097+-0.003	0.081+-0.003	0.047+-0.002
**chisq, df=5**	0.179+-0.004	0.231+-0.004	0.048+-0.002
**chisq, df=1**	0.458+-0.005	0.949+-0.002	0.051+-0.002

allele frequency 50%			
	**bartlett's**	**rank bartlett's**	**levene's**
**normal**	0.047+-0.002	0.047+-0.002	0.051+-0.002
**t, df=10**	0.11+-0.003	0.05+-0.002	0.05+-0.002
**t, df=5**	0.29+-0.005	0.052+-0.002	0.054+-0.002
**t, df=2**	0.907+-0.003	0.056+-0.002	0.046+-0.002
**chisq, df=15**	0.106+-0.003	0.092+-0.003	0.054+-0.002
**chisq, df=5**	0.185+-0.004	0.227+-0.004	0.049+-0.002
**chisq, df=1**	0.465+-0.005	0.907+-0.003	0.049+-0.002

Table S2

Type I error for a case when there is SNP effect which expalins 1% of total trait's variance

allele frequency 5%			
	**bartlett's**	**rank bartlett's**	**levene's**
**normal**	0.05+-0.002	0.05+-0.002	0.05+-0.002
**t, df=10**	0.105+-0.003	0.048+-0.002	0.047+-0.002
**t, df=5**	0.276+-0.004	0.053+-0.002	0.053+-0.002
**t, df=2**	0.887+-0.003	0.05+-0.002	0.055+-0.002
**chisq, df=15**	0.098+-0.003	0.086+-0.003	0.048+-0.002
**chisq, df=5**	0.188+-0.004	0.234+-0.004	0.052+-0.002
**chisq, df=1**	0.457+-0.005	0.973+-0.002	0.048+-0.002

allele frequency 10%			
	**bartlett's**	**rank bartlett's**	**levene's**
**normal**	0.052+-0.002	0.052+-0.002	0.05+-0.002
**t, df=10**	0.109+-0.003	0.047+-0.002	0.046+-0.002
**t, df=5**	0.281+-0.004	0.046+-0.002	0.047+-0.002
**t, df=2**	0.898+-0.003	0.05+-0.002	0.055+-0.002
**chisq, df=15**	0.094+-0.003	0.083+-0.003	0.048+-0.002
**chisq, df=5**	0.185+-0.004	0.232+-0.004	0.049+-0.002
**chisq, df=1**	0.468+-0.005	0.977+-0.001	0.053+-0.002

allele frequency 25%			
	**bartlett's**	**rank bartlett's**	**levene's**
**normal**	0.05+-0.002	0.05+-0.002	0.051+-0.002
**t, df=10**	0.108+-0.003	0.05+-0.002	0.049+-0.002
**t, df=5**	0.291+-0.005	0.053+-0.002	0.052+-0.002
**t, df=2**	0.907+-0.003	0.045+-0.002	0.042+-0.002
**chisq, df=15**	0.094+-0.003	0.082+-0.003	0.047+-0.002
**chisq, df=5**	0.187+-0.004	0.225+-0.004	0.049+-0.002
**chisq, df=1**	0.459+-0.005	0.947+-0.002	0.049+-0.002

allele frequency 50%			
	**bartlett's**	**rank bartlett's**	**levene's**
**normal**	0.045+-0.002	0.045+-0.002	0.049+-0.002
**t, df=10**	0.106+-0.003	0.05+-0.002	0.05+-0.002
**t, df=5**	0.295+-0.005	0.05+-0.002	0.049+-0.002
**t, df=2**	0.907+-0.003	0.054+-0.002	0.047+-0.002
**chisq, df=15**	0.098+-0.003	0.085+-0.003	0.049+-0.002
**chisq, df=5**	0.184+-0.004	0.234+-0.004	0.051+-0.002
**chisq, df=1**	0.452+-0.005	0.905+-0.003	0.049+-0.002

Table S3

Type I error for a case when there is SNP effect which expalins 5% of total trait's variance

allele frequency 5%			
	bartlett's	rank bartlett's	levene's
normal	0.049+-0.002	0.051+-0.002	0.05+-0.002
t, df=10	0.108+-0.003	0.05+-0.002	0.049+-0.002
t, df=5	0.269+-0.004	0.048+-0.002	0.046+-0.002
t, df=2	0.893+-0.003	0.049+-0.002	0.05+-0.002
chisq, df=15	0.098+-0.003	0.085+-0.003	0.05+-0.002
chisq, df=5	0.187+-0.004	0.232+-0.004	0.051+-0.002
chisq, df=1	0.464+-0.005	0.977+-0.002	0.05+-0.002

allele frequency 10%			
	bartlett's	rank bartlett's	levene's
normal	0.051+-0.002	0.052+-0.002	0.051+-0.002
t, df=10	0.112+-0.003	0.05+-0.002	0.051+-0.002
t, df=5	0.273+-0.004	0.05+-0.002	0.05+-0.002
t, df=2	0.902+-0.003	0.05+-0.002	0.052+-0.002
chisq, df=15	0.096+-0.003	0.081+-0.003	0.05+-0.002
chisq, df=5	0.184+-0.004	0.228+-0.004	0.051+-0.002
chisq, df=1	0.452+-0.005	0.977+-0.002	0.05+-0.002

allele frequency 25%			
	bartlett's	rank bartlett's	levene's
normal	0.049+-0.002	0.048+-0.002	0.049+-0.002
t, df=10	0.109+-0.003	0.052+-0.002	0.047+-0.002
t, df=5	0.282+-0.005	0.05+-0.002	0.053+-0.002
t, df=2	0.909+-0.003	0.048+-0.002	0.04+-0.002
chisq, df=15	0.095+-0.003	0.083+-0.003	0.048+-0.002
chisq, df=5	0.189+-0.004	0.235+-0.004	0.051+-0.002
chisq, df=1	0.449+-0.005	0.947+-0.002	0.05+-0.002

allele frequency 50%			
	bartlett's	rank bartlett's	levene's
normal	0.051+-0.002	0.05+-0.002	0.053+-0.002
t, df=10	0.105+-0.003	0.048+-0.002	0.049+-0.002
t, df=5	0.289+-0.005	0.05+-0.002	0.048+-0.002
t, df=2	0.912+-0.003	0.051+-0.002	0.044+-0.002
chisq, df=15	0.098+-0.003	0.086+-0.003	0.053+-0.002
chisq, df=5	0.186+-0.004	0.235+-0.004	0.05+-0.002
chisq, df=1	0.462+-0.005	0.906+-0.003	0.049+-0.002
