# Supplementary material for: Variance heterogeneity analysis for detection of potentially interacting genetic loci: method and its limitations
Source: BMC Genet. 2010 Oct 13;11:92. doi: 10.1186/1471-2156-11-92 (PMC2973850; doi:10.1186/1471-2156-11-92)
Supplement: Additional file 5 — Type I error for a case when genotype BB is tested against AB and AA. Type I error for 1df variance homogeneity tests when BB is tested against AA and AB when there is effect of SNP which explains 0%, 1%, and 5% of total trait's variance for different frequency of interacting allele (5%, 10%, 25% and 50%) and for different distribution of residual error (normal, three types of t and chi square distribution ). [file 1471-2156-11-92-S5.PDF]

Type I error for 1df variance homogeneity tests when BB genotype is tested against AA and AB in a case there is effect of SNP which explains 0%, 1%, and 5% of total trait's variance for different frequency of interacting allele (5%, 10%, 25% and 50%) and for different distribution of residual error (normal, three types of t and chi square distribution).

Table S1

Type I error for a case when there is no SNP

**B allele frequency 5%**			
	**bartlett's**	**rank bartlett's**	**levene's**
**normal**	0.052+-0.002	0.052+-0.002	0.05+-0.002
**t, df=10**	0.097+-0.003	0.052+-0.002	0.048+-0.002
**t, df=5**	0.173+-0.004	0.054+-0.002	0.048+-0.002
**t, df=2**	0.758+-0.004	0.06+-0.002	0.03+-0.002
**chisq, df=15**	0.084+-0.003	0.081+-0.003	0.047+-0.002
**chisq, df=5**	0.153+-0.004	0.211+-0.004	0.044+-0.002
**chisq, df=1**	0.409+-0.005	0.824+-0.004	0.04+-0.002

**allele frequency 10%**			
	**bartlett's**	**rank bartlett's**	**levene's**
**normal**	0.049+-0.002	0.049+-0.002	0.05+-0.002
**t, df=10**	0.101+-0.003	0.049+-0.002	0.049+-0.002
**t, df=5**	0.22+-0.004	0.051+-0.002	0.05+-0.002
**t, df=2**	0.836+-0.004	0.051+-0.002	0.04+-0.002
**chisq, df=15**	0.088+-0.003	0.078+-0.003	0.048+-0.002
**chisq, df=5**	0.176+-0.004	0.224+-0.004	0.048+-0.002
**chisq, df=1**	0.435+-0.005	0.899+-0.003	0.045+-0.002

**allele frequency 25%**			
	**bartlett's**	**rank bartlett's**	**levene's**
**normal**	0.05+-0.002	0.05+-0.002	0.05+-0.002
**t, df=10**	0.11+-0.003	0.051+-0.002	0.053+-0.002
**t, df=5**	0.255+-0.004	0.048+-0.002	0.048+-0.002
**t, df=2**	0.886+-0.003	0.05+-0.002	0.053+-0.002
**chisq, df=15**	0.096+-0.003	0.082+-0.003	0.05+-0.002
**chisq, df=5**	0.18+-0.004	0.232+-0.004	0.052+-0.002
**chisq, df=1**	0.446+-0.005	0.899+-0.003	0.048+-0.002

**allele frequency 50%**			
	**bartlett's**	**rank bartlett's**	**levene's**
**normal**	0.051+-0.002	0.05+-0.002	0.051+-0.002
**t, df=10**	0.109+-0.003	0.052+-0.002	0.051+-0.002
**t, df=5**	0.283+-0.005	0.051+-0.002	0.055+-0.002
**t, df=2**	0.904+-0.003	0.051+-0.002	0.046+-0.002
**chisq, df=15**	0.099+-0.003	0.087+-0.003	0.049+-0.002
**chisq, df=5**	0.187+-0.004	0.228+-0.004	0.048+-0.002
**chisq, df=1**	0.455+-0.005	0.901+-0.003	0.049+-0.002

Table S2

Type I error for a case when there is SNP effect which expalins 1% of total trait's variance

allele frequency 5%			
	**bartlett's**	**rank bartlett's**	**levene's**
**normal**	0.046+-0.002	0.046+-0.002	0.045+-0.002
**t, df=10**	0.096+-0.003	0.052+-0.002	0.049+-0.002
**t, df=5**	0.17+-0.004	0.052+-0.002	0.047+-0.002
**t, df=2**	0.752+-0.004	0.058+-0.002	0.034+-0.002
**chisq, df=15**	0.087+-0.003	0.086+-0.003	0.047+-0.002
**chisq, df=5**	0.16+-0.004	0.213+-0.004	0.047+-0.002
**chisq, df=1**	0.41+-0.005	0.823+-0.004	0.041+-0.002

allele frequency 10%			
	**bartlett's**	**rank bartlett's**	**levene's**
**normal**	0.05+-0.002	0.05+-0.002	0.054+-0.002
**t, df=10**	0.109+-0.003	0.051+-0.002	0.053+-0.002
**t, df=5**	0.216+-0.004	0.048+-0.002	0.044+-0.002
**t, df=2**	0.834+-0.004	0.05+-0.002	0.037+-0.002
**chisq, df=15**	0.094+-0.003	0.084+-0.003	0.051+-0.002
**chisq, df=5**	0.174+-0.004	0.224+-0.004	0.049+-0.002
**chisq, df=1**	0.438+-0.005	0.901+-0.003	0.044+-0.002

allele frequency 25%			
	**bartlett's**	**rank bartlett's**	**levene's**
**normal**	0.052+-0.002	0.051+-0.002	0.05+-0.002
**t, df=10**	0.11+-0.003	0.053+-0.002	0.052+-0.002
**t, df=5**	0.26+-0.004	0.052+-0.002	0.05+-0.002
**t, df=2**	0.883+-0.003	0.05+-0.002	0.052+-0.002
**chisq, df=15**	0.096+-0.003	0.084+-0.003	0.051+-0.002
**chisq, df=5**	0.18+-0.004	0.224+-0.004	0.049+-0.002
**chisq, df=1**	0.455+-0.005	0.904+-0.003	0.049+-0.002

allele frequency 50%			
	**bartlett's**	**rank bartlett's**	**levene's**
**normal**	0.049+-0.002	0.049+-0.002	0.051+-0.002
**t, df=10**	0.107+-0.003	0.049+-0.002	0.049+-0.002
**t, df=5**	0.284+-0.005	0.049+-0.002	0.047+-0.002
**t, df=2**	0.902+-0.003	0.051+-0.002	0.049+-0.002
**chisq, df=15**	0.095+-0.003	0.085+-0.003	0.05+-0.002
**chisq, df=5**	0.189+-0.004	0.237+-0.004	0.05+-0.002
**chisq, df=1**	0.463+-0.005	0.902+-0.003	0.053+-0.002

Table S3

Type I error for a case when there is SNP effect which expalins 5% of total trait's variance

allele frequency 5%			
	**bartlett's**	**rank bartlett's**	**levene's**
**normal**	0.048+-0.002	0.048+-0.002	0.049+-0.002
**t, df=10**	0.094+-0.003	0.049+-0.002	0.046+-0.002
**t, df=5**	0.167+-0.004	0.05+-0.002	0.044+-0.002
**t, df=2**	0.752+-0.004	0.062+-0.002	0.031+-0.002
**chisq, df=15**	0.088+-0.003	0.079+-0.003	0.046+-0.002
**chisq, df=5**	0.154+-0.004	0.208+-0.004	0.04+-0.002
**chisq, df=1**	0.407+-0.005	0.812+-0.004	0.041+-0.002

allele frequency 10%			
	**bartlett's**	**rank bartlett's**	**levene's**
**normal**	0.052+-0.002	0.053+-0.002	0.053+-0.002
**t, df=10**	0.102+-0.003	0.047+-0.002	0.044+-0.002
**t, df=5**	0.215+-0.004	0.044+-0.002	0.044+-0.002
**t, df=2**	0.84+-0.004	0.055+-0.002	0.04+-0.002
**chisq, df=15**	0.093+-0.003	0.085+-0.003	0.049+-0.002
**chisq, df=5**	0.176+-0.004	0.224+-0.004	0.045+-0.002
**chisq, df=1**	0.448+-0.005	0.894+-0.003	0.052+-0.002

allele frequency 25%			
	**bartlett's**	**rank bartlett's**	**levene's**
**normal**	0.048+-0.002	0.046+-0.002	0.05+-0.002
**t, df=10**	0.106+-0.003	0.05+-0.002	0.05+-0.002
**t, df=5**	0.26+-0.004	0.049+-0.002	0.05+-0.002
**t, df=2**	0.888+-0.003	0.05+-0.002	0.05+-0.002
**chisq, df=15**	0.099+-0.003	0.086+-0.003	0.049+-0.002
**chisq, df=5**	0.185+-0.004	0.23+-0.004	0.053+-0.002
**chisq, df=1**	0.448+-0.005	0.904+-0.003	0.05+-0.002

allele frequency 50%			
	**bartlett's**	**rank bartlett's**	**levene's**
**normal**	0.051+-0.002	0.052+-0.002	0.051+-0.002
**t, df=10**	0.102+-0.003	0.046+-0.002	0.048+-0.002
**t, df=5**	0.276+-0.004	0.048+-0.002	0.048+-0.002
**t, df=2**	0.904+-0.003	0.052+-0.002	0.046+-0.002
**chisq, df=15**	0.099+-0.003	0.087+-0.003	0.051+-0.002
**chisq, df=5**	0.184+-0.004	0.229+-0.004	0.052+-0.002
**chisq, df=1**	0.46+-0.005	0.906+-0.003	0.049+-0.002
